# Beyond Proximity: Utility-Based Access from Location-Based Services Data

**DOI:** 10.3390/ijerph191912352

**Published:** 2022-09-28

**Authors:** Gregory S. Macfarlane, Emma Stucki, Alisha H. Redelfs, Lori Andersen Spruance

**Affiliations:** 1 Civil and Construction Engineering Department, Brigham Young University, Provo, UT 84602, USA; 2 Public Health Department, Brigham Young University, Provo, UT 84602, USA

**Keywords:** accessibility, spatial analysis, location-based services data, community resources, parks, groceries, libraries

## Abstract

**Simple Summary:**

Proximity to community resources is often used as a benchmark in spatial public health analyses, but a measure that incorporates observed preferences leads to different policy interventions.

**Abstract:**

Understanding who in a community has access to its resources—parks, libraries, grocery stores, etc.—has profound equity implications, but typical methods to understand access to these resources are limited. Travel time buffers require researchers to assert mode of access as well as an arbitrary distance threshold; further, these methods do not distinguish between destination quality attributes in an effective way. In this research, we present a methodology to develop utility-based accessibility measures for parks, libraries, and grocery stores in Utah County, Utah. The method relies on passive location-based services data to model destination choice to these community resources; the destination choice model utility functions in turn allow us to develop a picture of regional access that is sensitive to: the quality and size of the destination resource; continuous (non-binary) travel impedance by multiple modes; and the sociodemographic attributes of the traveler. We then use this measure to explore equity in access to the specified community resources across income level in Utah County: the results reveal a discrepancy between which neighborhoods might be targeted for intervention using space-based analysis.

## 1. Introduction

Communities provide important resources to the people who live in them. These resources might include physical and economic resources—shared open space, libraries, commercial establishments, etc.—as well as less identifiable resources including a sense of membership and other forms of social capital [1]. Indeed, access to these resources is a primary reason why communities exist [2], as well as a long-motivating objective in transportation infrastructure planning [3].

Given the importance of these community resources, it is not surprising that so much scholarly attention has been paid to examining the spatial and socioeconomic variation in access to them [4,5]. What is surprising is the simplistic and arbitrary definition of many quantitative resource accessibility measures, in spite of the widespread availability of geographical information systems (GIS) software [6] and an understanding that proximity to a resource is not the only consideration in its use [7]. Individuals do not always shop at the nearest grocery store, nor do they necessarily perceive an 11-min walk to a park as meaningfully different from a 9-min walk. A measure of access that can incorporate travel impedance by multiple transportation modes alongside qualitative attributes of the resources in question would provide a better theoretical comparison to what people experience and observe in their own communities. This measure in turn may result in a different understanding of which groups have or do not have good access—and therefore in different policy interventions to resolve the access gap—than more traditionally used measures [6,8].

In this paper, we develop utility-based access measures to parks, grocery stores, and libraries in Utah County, Utah. These measures are based in econometric choice theory relating continuous multimodal travel impedance to attributes of the resource. The utility preferences are estimated on location-based services data obtained from a third-party commercial data aggregator. We then use the model estimates to construct a composite accessibility measure and examine potential discrepancies between this measure and a more common travel-time buffer measurement.

The paper begins with a discussion of previous findings relating access to community resources with social, health, and equity benefits. We then describe the methodology employed in this research, which makes use of novel third-party mobile device. A results section describes both the estimated choice models and a comparative analysis; the paper closes with a discussion of several limitations of the approach as well as associated opportunities for future research.

## 2. Literature Review

In this study, we have chosen to focus on three specific community resources that have robust histories of accessibility and spatial analysis: parks, grocery stores, and libraries. This section first discusses research developing and classifying various accessibility measures, followed by a discussion of previous attempts to measure access to the resources selected for this analysis.

### 2.1. Developing Access Measures

Accessibility is easily defined as the ability to reach useful destinations [4], but this ease in definition belies a wide array of potential quantitative descriptions. Dong et al. [7] present a helpful hierarchy of access measures, which we briefly summarize here.

Among the simplest measures of access is a so-called isochrone or buffer measure, which considers whether a person at position *i* traveling to a potential destination *j* is within a particular travel time threshold $t^{*}$. Using this measure, a person has access to the resource if $t_{ij}<t^{*}$. Sometimes it is possible to access multiple resources within this threshold, in which case a a cumulative opportunities measure can be defined as

(1) $$A_{i}^{\mathrm{isochrone}}=\sum_{j\in J}\delta_{ij},\;\mathrm{where}\;\delta_{ij}=\left\{\begin{array}{cc} 1, & \mathrm{for}\;t_{ij}\leq t^{*}\\ 0, & \mathrm{for}\;t_{ij}>t^{*} \end{array}\right.$$

Variations of this measure include elements like “number of grocery stores within 10 min” or “density of green space within 5 miles.” Further modifications might allow a measure to include the supply of the resource in addition to its spatial location [9]. Strengths of this method include its relative simplicity, but it has three central limitations. First, the threshold $t^{*}$ must be defined by the researcher for a specific value by a certain travel mode, and different definitions can have different policy outcomes [6]. Second, the binary nature of the measure contradicts a practical understanding of travel behavior: a four minute and fifty second trip is not functionally different from a five minute and ten second trip. Finally, the measure assumes that all options in the choice set *J* are of equivalent quality.

Extensions to this basic framework relax some of these constricting assumptions. A gravity-based accessibility measure

(2) $$A_{i}^{\mathrm{gravity}}=\sum_{j\in J}S_{j}*f\left(t_{ij},\beta \right)$$

considers the “size” of the destination $S_{j}$ as well as a continuous travel impedance function that decreases the impact of further destinations. The parameters β of this impedance function can be calibrated to match the observed trip length distribution of a survey or other data, or a basic distance decay function without parameters can be used. Additionally, if no other information on the “size” or attractiveness of the destinations is available, then $S_{j}=1$.

Activity-based or utility-based measures rely on location choice theory, where the probability of choosing a destination is a function of the destination’s attributes weighted against the travel impedance to reach it. The mathematical details of this measure are described below in Section 3.1, but the measure relies on understanding how the attributes of a destination $X_{j}$ affect the utility $V_{ij}$ of a person at origin *i* selecting that destination

(3) $$V_{ij}=X_{ij}\beta$$

where the marginal effect of the attributes are defined by a vector of estimable parameters β. One potential obstacle to developing utility-based accessibility measures has been obtaining sufficient data on which to estimate these utility preference parameters. High-quality household surveys that reveal activity locations are most commonly used for this purpose in general travel demand modeling, but such surveys typically group many infrequent discretionary trips into catch-all categories [10].

In the last several years, however, various commercial data products developed from mobile device and location-based services (LBS) data have entered common use in transportation planning activities. Applications or websites that serve mobile content based on a user’s location will log this location information and sell the data to commercial third-party aggregators. These aggregators in turn will weight and anonymize the data before selling the prepared datasets to transportation planning agencies. These LBS datasets typically contain vehicle or person flows between spatially defined zones, sometimes segmented by inferred transportation mode, time of day, day of week, or imputed trip purpose. These datasets have been shown to accurately reflect visits to recreation areas and other land uses [11], and are becoming a common part of transportation planning practice [12,13]. In recent years, researchers have begun developing methods to estimate destination choice models (and their related utility parameters) from passive data. Zhu and Ye [14] developed a method to estimate a destination choice model for taxi trips in Shanghai, relying on the scale of the GPS dataset to estimate a robust model. Macfarlane et al. [15] use location-based services data for park visitors in Alameda County, California to estimate a destination choice model, and then apply that model to examine utility-based park accessibility and equity.

### 2.2. Access to Parks, Grocery Stores, and Libraries

Parks and other open spaces are frequently understood to provide mental and physical health benefits to the members of the community who use them [16], but specific evidence of a link between access and these benefits is somewhat mixed, perhaps due to a wide variety of accessibility measures used in various studies [17]. Most use some form of isochrone-based measure. For example, Neusel Ussery et al. [18] developed a county-level green space density measure for the entire United States based on the percentage of developed green space in each county. A popular measure called ParkScore [19] uses the share of a population that lives within a 10-min walk of a park to provide a metropolitan-level accessibility score. Some studies have shown that metropolitan areas with a higher ParkScore have better health outcomes [20], but this finding has not been satisfactorily reproduced for neighborhoods within a metropolitan region. Kaczynski et al. [21] developed ParkIndex, a measure that gives extra weight to neighborhoods near high-quality parks by incorporating park choice preferences determined from a user survey; some of the usefulness of this measure is limited by only weighting neighborhoods within 1 mile of a park rather than being applied continuously across the region as a utility-based access measure. Macfarlane et al. [8] constructed a utility-based access to parks measure derived from an earlier park choice survey [22], and showed a positive relationship between this measure and health outcomes that does not appear to exist when using the ParkScore isochrone access measure.

The accessibility of grocery stores to low-income or other disadvantaged communities has been a similarly frequent topic in the academic literature; both in terms of identifying the existence of so-called “food deserts” as well as correlating these deserts with measures of well-being. The U.S. Department of Agriculture (USDA) defines food deserts for their own purposes as low-income census tracts where a certain threshold of people live more than a mile from the nearest grocery store, or a shorter threshold if automobile ownership is low [23]. Most other researchers have adopted similar definitions of access. For example, Morland et al. [24] use the number of grocery stores in the same census tract, Algert et al. [25] used the share of households within 0.8 kilometers of a store, and Hamidi [26] uses the USDA measures directly. In conflict with these simplistic definitions are a number of studies suggesting that the nearest grocery store is not necessarily where people—including low-income people—obtain their food [27,28,29]. Wood and Horner [30] addressed this shortcoming by considering a gravity-derived accessibility measure, weighting the number of opportunities against a continuous travel time function. Other more recent research has suggested that what matters is not home accessibility as much as location of a store within a time-space construction of a person’s daily activities [31,32].

Libraries provide important educational and social opportunities for community members through computer facilities, reference materials, and special programs [33,34]. Libraries can also be used to enhance physical and emotional well-being in a community through public initiatives [35]. Though perhaps not as commonly studied as either parks or libraries, a few recent efforts have examined the spatial distribution of libraries and socioeconomic disparities in access. Allen [36] measured the gap in travel time to the nearest library by car and by public transit, showing that transit-dependent communities were considerably disadvantaged. Cheng et al. [37] applied travel time thresholds to examine the share of communities in Hong Kong that lacked access. Guo et al. [38] also measured library access disparities in Hong Kong, using two different travel-time focused measures. None of the measures we could find considered other attributes of the library beyond its proximity, even though these additional features play a strong role in the library’s role in community building [34].

Certainly there are other community resources that warrant consideration; Ermagun and Tilahun [39] consider a multiple-resource gravity accessibility measure that includes schools, jobs, and hospitals in addition to the three that have been used here. Churches, museums, or various other facilities might be relevant elements in shaping the quality of life in a community. Regardless of what resources are selected, it is clear that existing accessibility practice considers spatial proximity as paramount, and quality of the destination as secondary. Further, travel times by particular modes are the default measure rather than holistic, multimodal travel impedance measures. Using utility-based measures for both travel impedance and for the accessibility measure might provide a more complete picture of who can and who cannot access community resources in a region.

## 3. Methods

In this section, we present a method to estimate utility-based access to community resources in Utah County, Utah.

### 3.1. Modeling Framework

In a destination choice modeling framework [27], an individual at origin *i* considering a destination *j* from a set of possible destinations *J* has a choice probability

(4) $$P_{ij}=\frac{e^{V_{ij}}}{\sum_{j^{\prime }\in J}e^{V_{ij^{\prime }}}}$$

where $V_{ij}$ is a linear-in-parameters function representing the utility of destination *j*. The destination utility consists of two basic elements:

(5) $$V_{ij}=\beta t_{ij}+X_{j}\gamma$$

where $t_{ij}$ is a measure of the travel impedance between *i* and *j*, $X_{j}$ is a vector of attributes of destination *j*, and β,γ are estimated parameters relating the travel impedance and the destination attributes to the utility. These parameters may be estimated by maximum likelihood given sufficient observational data.

The logarithm of the denominator of the choice probability given in Equation (4) is a quantity called the *logsum* and represents the total value—or accessibility *A*—of the choice set for individual *i* [4,40]

(6) $$A_{i}=\log\left(\sum_{j^{\prime }\in J}e^{V_{ij^{\prime }}}\right)+C$$

where *C* is an unknown constant resulting from the fact that the utility represented in Equation (5) is not absolute, but rather relative to the utilities of the other options. The difference in logsum values between two different origin points could be compared to determine which location has “better” accessibility to the destinations in question, based on the elements included in Equation (5). Accessibility might be improved by lower travel impedance, or by improved amenities, or even by simply having more options available.

These other elements include attributes of the community resource relevant to the destination choice problem: the size of the resource, amenities available, the price of goods on sale, etc. Each of these variables has an importance weighted against the travel impedance $t_{ij}$, which might take various forms depending on the data available and the destination resource in question.

Simple measures such as the highway travel time or the walk distance might be more or less appropriate for particular resources. Another option commonly used in travel demand models is actually the logsum of a *mode* choice model with the utility of choosing each mode given by a set of utility equations. In this study, we adopt generic mode choice utility equations

$$\begin{array}{cc} V_{ij\mathrm{auto}} & =-0.028*(t_{ij\mathrm{auto}})\\ V_{ij\mathrm{transit}} & =-4-0.028*\left(t_{ij\mathrm{transit}}\right)-0.056*\left(wt_{ij}\right)-0.372*\left(at_{ij}\right)\\ V_{ij\mathrm{walk}} & =-5-0.028*\left(t_{ij\mathrm{walk}}\right)-1.12*\left(d_{ij}|d_{ij}<1.5\right)-5.58*\left(d_{ij}|d_{ij}\geq 1.5\right) \end{array}$$

where $t_{ij}$ is the travel time in minutes from *i* to *j* by each mode (including only in-vehicle time for transit), wt is the transit wait and transfer time, at is the time to access and egress transit by walking, and $d_{ij}$ is the walking distance in miles. The walking distance uses two different utility parameters depending on whether the walking distance is greater than 1.5 miles. The travel impedance logsum between *i* and *j* is then

(7) $$MCLS_{ij}=\log(\exp\left(V_{ij\mathrm{auto}}\right)+\exp\left(V_{ij\mathrm{transit}}\right)+\exp\left(V_{ij\mathrm{walk}}\right))$$

### 3.2. Data

**Figure 1:**
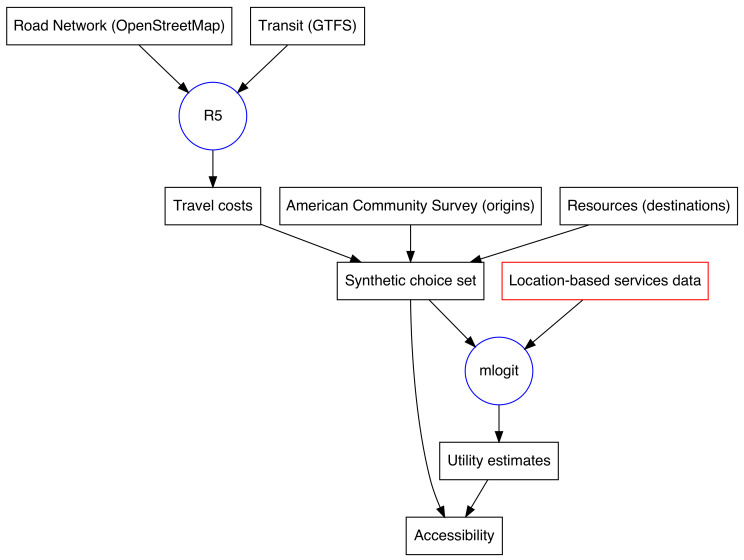
Diagram of the data assembly process.

Figure 1 presents a schematic of the process to calculate the accessibility logsum for a particular region. Though the remainder of this section provides detail on each step and data input, a high-level overview is perhaps useful here. First, the American Community Survey provides information on the “origins” or residence neighborhoods in the region of study. Manual data collection efforts or other methods provide information on the community resources under study, or the “destinations.” Routing software (R5) generates a matrix of travel costs between pairs of neighborhoods and resource locations using highway networks from OpenStreetMap and transit service timetables. These three data sets (origin information, destination information, and travel costs) are then combined into a “synthetic” choice set representing possible activity locations for people in each neighborhood. Location-based services data from a commercial provider reveals which potential destination was actually chosen, information which feeds an econometric choice model that estimates choice utility parameters. Finally, these utility parameters can then be re-applied to the choice set—or a new dataset representing future conditions or even a different region—to calculate utility-based accessibility measures.

Utah County, Utah, is among the fastest-growing urbanized regions in the United States, with formerly agrarian areas and open rangeland being converted to predominately suburban built environments. The population and economic center of the county is in Provo and Orem, home to two large universities (Brigham Young and Utah Valley), but the most rapid development in commercial and residential terms has been in communities north of Utah Lake, between Provo and Salt Lake City to the north. Interstate 15 travels the spine of the county, and a commuter rail system travels multiple times a day between Provo and Salt Lake City with stations in Orem, American Fork, and Lehi. A bus rapid transit (BRT) system connects the universities, two commuter rail stations, and the densest portions of Provo and Orem, and other local bus services operate in other communities in the region. Table 1 presents descriptive statistics of the block groups—a Census-defined geography between 600 and 3000 people, and the smallest geography at which aggregate demographic statistics are generally available—in Utah County obtained from the 2015–2019 American Community Survey (ACS) using the tidycensus package for R [41].

#### 3.2.1. Resource Data

**Figure 2:**
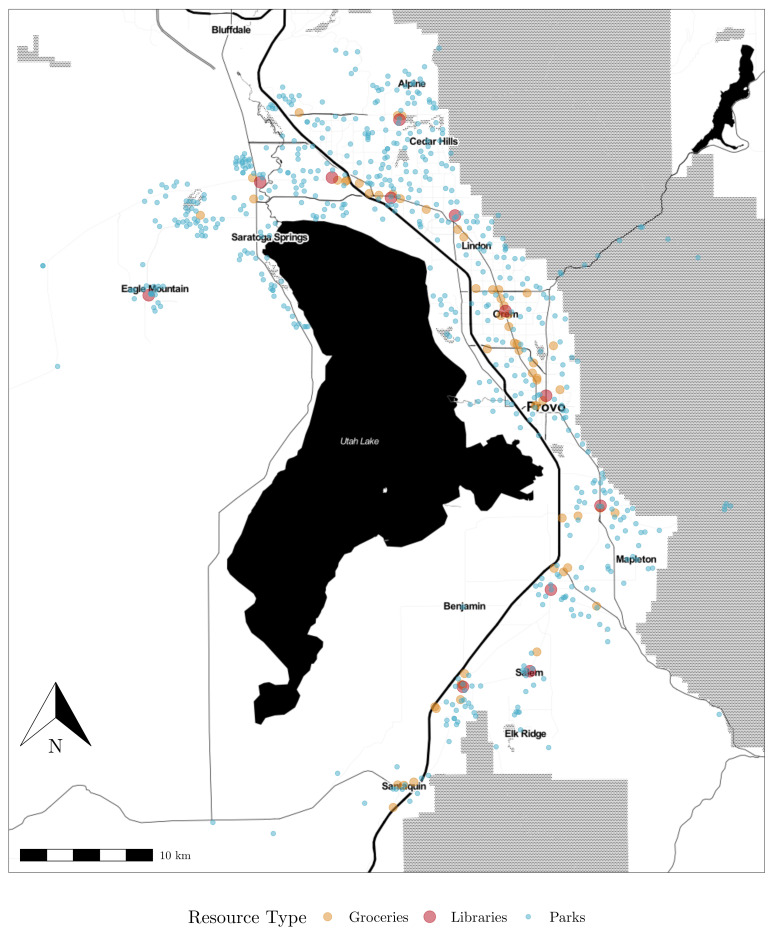
Location of community resources in Utah County.

Figure 2 shows the locations of three types of community resources in Utah County: parks, grocery stores, and libraries. For each resource, an initial list of resources and elementary attributes was obtained by executing a relevant query to OpenStreetMap (OSM).

Public parks and their attributes retrieved from OSM were verified and corrected using aerial imagery and some site visits. The attributes included the size of the park in acres, whether the park includes a playground, and whether the park includes facilities for volleyball, basketball, and tennis. The constructed dataset includes 582 attributed parks.

Grocery stores were retrieved from OSM and validated using internet resources and site visits. The complete Nutritional Environment Measures Survey (NEMS-S) [42] was collected for each store, but this preliminary analysis only includes cursory information on the stores including whether the store is a convenience store or some other non-traditional grocery, whether the store includes a pharmacy or other non-food merchandise, and the number of registers as a measure of the store’s size. The constructed dataset includes 58 stores.

Libraries were retrieved from OSM, and validated using library websites and some site visits. The initial query returned university libraries and other specialty resources; though some of these libraries are open to those outside the university community, these were removed so that the resource list only includes libraries generally catering to the general public. The amenities available include whether the library offers additional classes and programs, and whether the library includes genealogical or family history resources. The square footage of the library was estimated from online mapping services. Other variables discussed in the literature such as the availability of computers and public wi-fi networks were present in every library and therefore cannot be included in the destination utility equations.

#### 3.2.2. Mobile Device Data

Macfarlane et al. [15] present a method for estimating destination choice models from such data, which we repeat in this study. We provided a set of geometric polygons for each park, grocery store, and library to StreetLight Data, Inc., a commercial aggregator. StreetLight Data in turn provided data on the number of mobile devices observed in each polygon grouped by the inferred residence block group of those devices during summer and fall 2019. We then created a simulated destination choice estimation dataset for each community resource by sampling 10,000 block group—resource “trips” from the StreetLight dataset. This created a “chosen” alternative; we then sampled ten additional resources at random (each simulated trip was paired with a different sample) to serve as the non-chosen alternatives. Random sampling of alternatives is a common practice that results in unbiased estimates, though the standard errors of the estimates might be larger than could be obtained through a more carefully designed sampling scheme [43].

#### 3.2.3. Travel Times

Once the choice, alternatives, and attributes of the alternatives have been defined, the last element of the choice utility is the travel impedance between each block group and each resource. Using the r5r R interface [44] to the R5 routing algorithm, we estimated the highway drive travel time, the walking route time, and the transit travel time for trips departing on 26 April 2022 at 8 AM. The time and date are most relevant for the transit path builder in R5, which uses detailed transit path information stored in the Utah Transit Authority GTFS feed file for Spring and Summer 2022. The transit path contains separate measures of the total travel time, the time in the transit vehicle, transfer time, and access/egress time, allowing us full use of the mode choice utility equations and resulting logsum described in Equation (7). We limited valid paths to those involving less than 10 km of walking and 2 h of total travel time.

For groceries and libraries, we queried the shortest time path on each mode from the population-weighted block group centroid to the centroid of the grocery store or library polygon. Because some parks in the dataset can be relatively large and the centroid might be far from the park access or use point, we instead sampled points along the boundary of the park polygon, and queried the shortest time path by each mode to the nearest boundary point.

## 4. Results

### 4.1. Destination Choice Models

Using the simulated trip choices assembled from the location-based services data, we estimate destination choice models with the mlogit package for R [45,46].

Table 2 presents the model estimation results for five different specifications of park destination choice. The “Car” model includes only the network travel time by car as a predictor of park choice; the “MCLS” model similarly contains only the mode choice logsum as an impedance term. The signs on the coefficient indicate that people are more likely to choose parks with lower car distance or higher multi-modal access, all else equal. The “Attributes” model includes only information on the park attributes including size and amenities. On balance, people appear attracted to larger parks and parks with playgrounds, while somewhat deterred by various sports facilities. The “All” models include both the relevant travel impedance term as well as destination attributes.

For most block group-park pairs, the transit and walk travel costs are sufficiently high that choosing these travel modes is unlikely. As a result, the mode choice logsum is highly collinear with the car travel time. Nevertheless, there are small differences between the models with the different impedance terms. Using a non-nested likelihood statistic test presented by Horowitz [47], we cannot reject the null hypothesis that the two “All” models have equivalent likelihood (*p*-value of 0.195), and therefore cannot infer that the mode choice logsum is a marginally better estimator of park choice than the vehicle travel time alone.

Table 3 presents the model estimation results for the grocery store models. As with the parks models in Table 2, the most predictive model contains both a travel impedance term and attributes of the destination grocery store. The number of registers suggests that people prefer larger stores, all else equal; ethnic markets, convenience stores, and other facilities are less preferred while stores with pharmacies and other merchandise (clothes, home goods, etc.) attract visitors. The ratio of the drive time and convenience store coefficients suggests that on average, people are willing to drive 4.97 min to reach a store that is not a convenience store. In terms of the travel impedance, there is again not a sufficiently large gap in the model likelihoods to reject that the mode choice logsum and the drive time are equivalent predictors of grocery store choice.

Table 4 presents the model estimation results for the library destination choice models. As with parks and grocery stores, both travel impedance and destination attributes are significant predictors of library choice. The strength of the attributes vector is somewhat surprising, because virtually all libraries in the data set offer the same set of basic amenities other than the size of the facility. Further, each municipality in Utah County operates its own library rather than having a system of interconnected library branches as might be typical in larger cities or other regions. As with grocery stores and parks, there is no significant difference between the prediction power of the mode choice logsum versus the car travel time.

### 4.2. Accessibilities

Using the results of the “All - Logsum” models estimated for each community resource in the last section, we calculate the total utility-based accessibility measure for each block group in Utah County. For comparison to a more traditional measure, we also created buffer-based accessibility terms where a block group has “access” to a grocery store if there is one within a 5-min drive, a park if there is one within a five-minute walk, and a library if there is one within a ten-minute drive.

**Figure 3:**
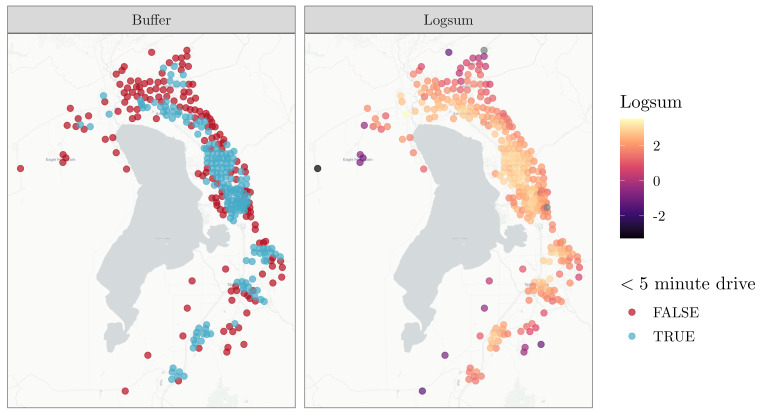
Spatial comparison of grocery access threshold versus logsum value.

Figure 3 spatially presents the difference between the buffer-based measure and the logsum-based measure. The two measures largely show the same basic shape: block groups along the spine of the county tend to have binary access in the buffer and also have a higher logsum value. The difference is at the margins, where the discontinuity of the buffer measure is replaced by a smoother access surface, more spatially reflective of what people are likely to experience.

**Figure 4:**
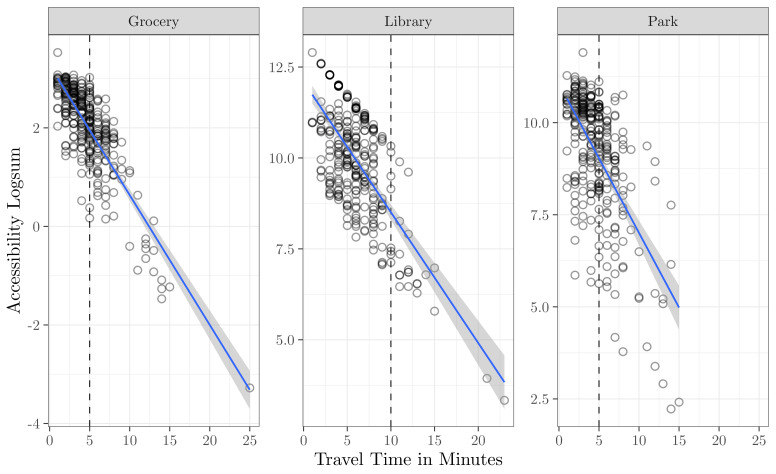
Relationship between travel time and logsum access value for block groups in Utah County. Travel time thresholds shown as dashed lines, with best fit regresssion line added for context.

The potential for the buffer measure to oversimplify the accessibility problem is further illustrated in Figure 4. This figure shows the utility-based accessibility logsum calculated using the mode choice logsum as an impedance term against the travel time in minutes (drive time for grocery stores and libraries; walk time for parks), for block groups in the study region. It is clear that for all three land uses, lower travel time is significantly correlated with higher accessibility. However, for block groups with equivalent travel time to a particular community resource, the accessibility logsum value varies substantially. Even for block groups along the buffer—where a small change in travel time might place a block within or without the buffer—the variance in accessibility logsum appears to be almost as large as the variance in the travel time. This variance in accessiblity logsum might be due to a travel time differential between drive, walk, and transit modes captured in the mode choice logsum, or it could also be because the resources available near the set of block groups have substantial variance in their amenities. Being near a single poor-quality grocery store is not the same thing as being near multiple high-quality groceries, and the logsum value can capture this variance in its construction.

### 4.3. Spatial Distribution

In this analysis, we estimate that 18,750 households live in block groups outside the boundary of all three resource buffers: 10-min drive for a library, 5-min drive for a grocery store, and 5-min walk for a park. Of these, 1648 make less than $35,000 per year. At the same time, only 33,814 households live in block groups that are beneath the regional mean utility-based access to all three resources; that is, they have less-than the regional average access to grocery stores, and to libraries, and to parks. Of these households, 3073 are similarly low-income. Perhaps more importantly, the overlap between the households in *both* groups is not very high: only 9405 households live in block groups with low access determined by both buffers and by accessibility logsum, 739 of which are low-income households.

## 5. Discussion

The results presented in the previous section suggest that professionals and academics must understand the implications of accessibility definitions when conducting spatial analysis of community resources. This study does not attempt to correlate the accessibility measure it presents with measures of nutrition, health, or numerous other potential covariates. However, it is not difficult to forsee how the results of such an analysis could change substantially based on whether a binary distance-based or utility-based definition is used. Further, the lack of spatial agreement on which people in the community have access or do not have access implies that policy interventions constructed based on distance alone may target neighborhoods that actually have good access from a more holistic perspective.

Surely there are limitations to the specific methods developed in this study. The location-based services data reveals the likely home location of devices observed within a geographic polygon, within some measurement error. It cannot tell us whether the device holder actually accomplished the assumed activity; that is, there may be a reason why a device was observed near a library even though the person did not actually patronize the library. Additionally, the method we use to compile the estimation dataset presumes that the choice to make a trip to the community resource has already been made. Though it can suggest how the accessibility of a neighborhood to these resource would improve were transportation impedance decreased or the resources expanded or improved, it cannot tell us how many more people might take advantage of the resource in that case.

In this research, all simulated trips were grouped into a single pooled model for analysis. This implies that the effect of amenities and travel impedance on destination choice is similar for all neighborhoods. A segmented model where, for example, low-income block groups and high-income block groups were estimated separately could allow for flexibility in these estimates and reveal differences in preferences among residents of the different neighborhoods. Some neighborhoods might show a particular preference for access utilities by transit, or for specific park amenities. A latent class choice model [48] would go further in potentially informing which demographic variables are meaningful in defining possible data segmentation schemes. It may be also be possible to estimate the models using a synthetic population with statistical resampling instead of block group-level aggregate demographic measures.

A necessary assumption made when constructing the estimation dataset is that people experience access from their home neighborhood. This may not always be true; for instance, people may choose to shop at grocery stores or visit libraries that are near their workplace, or that are between their homes and some other frequent destination. Methods to account for access and destination choices experienced at other points in the day would be a useful and interesting extension. Similarly, we assumed that the distance between a home and a community resource was represented by the distance between the block group centroid and the resource. For some block groups in less dense areas of the county, the error in measured travel time between the block group centroid and the actual home location might be larger than the total travel time. It might be possible to simulate home locations within each block group and use those locations in the travel time calculations. Alternatively, it might be possible to estimate the model using block group data as in this study but apply the model at a more fine resolution (e.g., block) when investigating accessibilities and conducting policy analyses.

This paper presents preliminary model estimates using plausible destination choice utility values. Several additional variables might be further explored, particularly in regards to the grocery resources. The NEMS-S survey is a highly detailed picture of the offerings of a particular grocery store, including information on the availability of relatively healthier or fresher foods and their prices. This study was only able to explore a few key size variables, but a deeper investigation into grocery store amenities and offerings preferences—and how they might influence a collective understanding of nutrition access more broadly—is needed.

## 6. Conclusions

This paper developed accessibility-based measures of accessibility to three types of community resource: parks, grocery stores, and libraries. These metrics were informed by observing trips to specific facilities in mobile device data, allowing the measures to incorporate attributes of the resource as well as attributes of the journey there. The computed measures are fundamentally different from buffer-based measures more commonly used to inform spatial policy analysis.

Ultimately, the purpose of any accessibility measure to a community resources is to enable a subsequent analysis of some metric of well-being. Macfarlane et al. [8] suggest that a utility-based access to parks measure is more predictive of physical health outcomes than a buffer-based measure. Is this true for more community resources? Would using a more subtle or nuanced measure of access to libraries help in understanding a link between community form and social isolation or mental health? A key benefit of this method is that it provides a way to evaluate the benefit of investments in resources against the benefits of investing in the transportation system. Will a community benefit more from a new grocery store nearby, or expanded options at an existing grocery store, or from improving bike or bus connections to that existing store? An examination of this question is left for future research, but this paper presents a method for how this could be approached.

## Figures and Tables

**Table 1:** Block Group Summary Statistics.

	Pct. Missing	Min	Mean	SD	Median	Max
Density: Households per square kilometer	0	0.00	558.95	660.05	394.68	4747.20
Income: Median block group annual income ($US)	2	20,588.00	80,309.14	31,030.52	77,099.00	196,458.00
Low Income: Percent of households making less than $35k	1	0.00	16.58	13.37	12.67	70.36
High Income: Percent of households making more than $125k	1	0.00	22.96	17.13	19.15	92.31
Children: Percent of households with children under 6	1	0.00	24.17	12.33	22.13	84.62
Black: Percent of population who is Black	0	0.00	0.45	0.95	0.00	7.37
Asian: Percent of population who is Asian	0	0.00	1.44	2.30	0.49	20.34
Hispanic: Percent of population who is Hispanic *	0	0.00	11.64	10.57	8.64	62.11
White: Percent of population who is White	0	32.84	82.56	11.88	84.25	100.00

* Hispanic indicates Hispanic individuals of all races; non-Hispanic individuals report a single race alone.

**Table 2:** Park Destination Choice Utilities.

	Car	MCLS	Attributes	All-Car	All-Logsum
Drive time	−0.286 (−93.013) **			−0.267 (−67.263) **	
Mode Choice Logsum		10.203 (93.013) **			9.547 (67.263) **
log(Acres)			1.317 (77.828) **	1.307 (45.585) **	1.307 (45.582) **
Playground			4.574 (34.228) **	4.467 (30.248) **	4.466 (30.247) **
Volleyball			−0.344 (−8.989) **	−0.555 (−9.379) **	−0.555 (−9.379) **
Basketball			−0.665 (−15.643) **	−0.508 (−7.024) **	−0.508 (−7.024) **
Tennis			−0.566 (−13.515) **	−0.881 (−14.706) **	−0.881 (−14.708) **
Num.Obs.	9119	9119	9119	9119	9119
Log Likelihood	−8945.6	−8944.8	−11,954.7	−4603.5	−4603.2
McFadden Rho-Sq	0.591	0.591	0.453	0.789	0.789

t-statistics in parentheses. ** *p* < 0.01.

**Table 3:** Grocery Destination Choice Utilities.

	Car	MCLS	Attributes	Size	All-Car	All-Logsum
Drive time	−0.251 (−94.328) **				−0.270 (−82.644) **	
Mode Choice Logsum		8.972 (94.329) **				9.643 (82.645) **
Convenience Store			−2.231 (−12.434) **	−1.520 (−8.412) **	−1.343 (−6.987) **	−1.343 (−6.987) **
Other non-standard			−2.224 (−15.998) **	−1.618 (−11.535) **	−1.418 (−9.430) **	−1.418 (−9.429) **
Has pharmacy			0.603 (20.852) **	0.359 (10.659) **	0.330 (7.848) **	0.330(7.850) **
Ethnic market			−1.639 (−18.212) **	−0.976 (−10.569) **	−0.887 (−9.080) **	−0.887 (−9.082) **
Has other merchandise			1.495 (51.750) **	0.791 (21.345) **	0.909 (19.288) **	0.909 (19.291) **
Number of registers				0.071 (44.314) **	0.087 (40.073) **	0.087 (40.074) **
Number of self-checkout				0.026 (13.475) **	0.020 (8.207) **	0.020 (8.206) **
Num.Obs.	10,000	10,000	10,000	10,000	10,000	10,000
Log Likelihood	−14,256.7	−14,257.0	−20,199.6	−19,031.4	−10,542.9	−10,542.9
McFadden Rho-Sq	0.405	0.405	0.158	0.206	0.560	0.560

t-statistics in parentheses. ** *p* < 0.01.

**Table 4:** Library Destination Choice Utilities.

	Car	MCLS	Attributes	All-Car	All-Logsum
Drive time	−0.303 (−91.448) **			−0.313 (−72.838) **	
Mode Choice Logsum		10.814 (91.447) **			11.195 (72.836) **
Offers Classes			−0.471 (−11.662) **	−0.819 (−12.050) **	−0.819 (−12.051) **
Genealogy Resources			−0.831 (−30.213) **	−0.867 (−20.844) **	−0.867(−20.840) **
log(Square Footage)			1.114 (79.831) **	1.219 (44.555) **	1.219 (44.552) **
Num.Obs.	10,000	10,000	10,000	10,000	10,000
Log Likelihood	−11,198.0	−11,197.5	−17,667.4	−9389.7	−9389.6
McFadden Rho-Sq	0.533	0.533	0.263	0.608	0.608

t-statistics in parentheses. ** *p* < 0.01.

## Data Availability

The code used for this study, including code to obtain and construct the open data sets employed herein, is available on GitHub at https://github.com/byu-transpolab/Community_Resources_2021, accessed on 1 May 2022. The location-based services data is obtained under commercial license and cannot be distributed.
